# Endovascular Stent-Graft Repair of True and False Aneurysms of the Splenic Artery

**DOI:** 10.3390/jcm13102802

**Published:** 2024-05-09

**Authors:** Ottavia Borghese, Angelo Pisani, Antonio Luparelli, Simona Sica, Fabrizio Minelli, Tommaso Donati, Yamume Tshomba

**Affiliations:** 1 Unit of Vascular Surgery, Fondazione Policlinico Universitario Gemelli IRCCS, 00168 Rome, Italy; antonioluparelli10@gmail.com (A.L.); simonasica1@gmail.com (S.S.); fabrizio.minelli@policlinicogemelli.it (F.M.); tommaso.donati@policlinicogemelli.it (T.D.); 2 Post Doctoral School Angio-Cardio-Thoracic Pathophysiology and Imaging, Sapienza University, 00185 Rome, Italy; angelo.pisani@ymail.com; 3 Unit of Vascular Surgery, Department of Cardiovascular Sciences, Fondazione Policlinico Universitario Gemelli IRCCS-Università Cattolica del Sacro Cuore, Largo Agostino Gemelli, 8, 00168 Rome, Italy

**Keywords:** covered stents, splenic artery aneurysm, rupture, stent graft endovascular procedure

## Abstract

Objective: In treatment of aneurysms (SAAs) and pseudoaneurysms (SAPs) of the splenic artery, endovascular coil embolization is the approach most commonly used as it is minimally invasive and safe. However, it carries a significant rate of primary failure (up to 30%) and might be complicated by splenic infarction. The use of stent grafts might represent a valuable alternative when specific anatomical criteria are respected. We report a comprehensive review on technical and clinical outcomes achieved in this setting. **Methods:** We performed a comprehensive review of the literature through the MedLine and Cochrane databases (from January 2000 to December 2023) on reported cases of stenting for SAAs and SAPs. Outcomes of interest were clinical and technical success and related complications. The durability of the procedure in the long-term was also investigated. **Results:** Eighteen papers were included in the analysis, totalling 41 patients (n = 20 male 48.8%, mean age 55.5, range 32–82 years; n = 31, 75.6% SAAs). Mean aneurysm diameter in non-ruptured cases was 35 mm (range 20–67 mm), and most lesions were detected at the proximal third of the splenic artery. Stent grafting was performed in an emergent setting in n = 10 (24.3%) cases, achieving immediate clinical and technical success rate in 90.2% (n = 37) of patients regardless of the type of stent-graft used. There were no procedure-related deaths, but one patient died in-hospital from septic shock and n = 2 (4.9%) patients experienced splenic infarction. At the last available follow-up, the complete exclusion of the aneurysm was confirmed in 87.8% of cases (n = 36/41), while no cases of aneurysm growing nor endoleak were reported. None of the patients required re-intervention during follow-up. **Conclusions:** When specific anatomical criteria are respected, endovascular repair of SAAs and SAAPs using stent grafts appears to be safe and effective, and seems to display a potential advantage in respect to simple coil embolization, preserving the patient from the risk of end-organ ischemia.

## 1. Introduction

Splenic artery aneurysms (SAAs) account for about 60–70% of overall cases of aneurysms involving the visceral arteries, and are the most commonly encountered following those affecting the aorta and iliac vessels [1].

True splenic artery aneurysms represent a focal dilation of the artery with a diameter greater than 50% compared to the normal vessel diameter.

SAAs involve all three layers of the vessel wall (intima, media, and adventitia), while splenic artery pseudoaneurysms (SAPs) affect only one or two layers and develop following a tear in the intima with subsequent periarterial hematoma formation.

Risk factors for SAAs include advanced age, female gender, atherosclerosis, portal hypertension, liver transplantation, pregnancy, and connective tissue disorders (Marfan or Ehler–Danlos syndrome). Conversely, SAPs occur on a background of infection, trauma or iatrogenic injuries, peptic ulcer disease, or acute and chronic pancreatitis as a consequence of the disintegration of elastin fibres of the vessels’ wall by pancreatic enzymes [2].

Both lesions remain mostly asymptomatic, but rupture with limited gastrointestinal haemorrhage, hematemesis, haematochezia, haemobilia, or severe hypovolemic shock may be a potentially life-threatening event and represents the initial presentation in about 2–10% of all SAAs, with an increased risk (between to 76 and 83%) in symptomatic patients [3].

Interventional treatment of SAPs is always mandatory regardless their size, as they display relatively rapid growth rates and an increased risk of rupture when compared to SAAs in the same location. Conversely, SAAs should be repaired when they reach >30 mm in maximal diameter or regardless their size if detected in women of child-bearing age, in patients whose aneurysm demonstrates interval growth >0.5 cm/year, in those undergoing liver transplantation, or in those affected with portal hypertension [4].

Until the last decade, open surgical repair was the mainstay of therapy, and could include artery ligation with or without revascularization or end-organ resection, with associated mortality rates ranging between 5 and 25% [5,6].

Endovascular therapy with coil embolization is currently the first-line treatment, even though it carries a significant rate of primary failure (up to 30% of cases) as persistent sac perfusion, aneurysm recanalization, or coil migration can occur [7]. Additionally, this approach excludes distal circulation, which can compromise organ function; it has been reported that coil embolization might complicate with splenic infarction in up to 40% of the patients, leading to prolonged hospitalization, splenectomy, or percutaneous drainage for the treatment of abscesses or refractory pain [6,7,8].

In this setting, endovascular SAAs/SAPs’ repair using stent-graft could potentially minimize the risk of end-organ infarction, allowing for, at the same time, the exclusion of the aneurysm. Several reports have emerged describing this technique as a valuable alternative that compensates for the disadvantages of embolization [9], even though their use in infection is controversial, and not all patients are eligible for this approach as specific anatomic features are required and the stent-graft delivery might be hampered by the vessels’ tortuosity.

The currently available data come from individual cases or small series with only short-term results, and the body of the literature lacks a summary of the available knowledge about the immediate and long-term clinical and technical outcomes achieved in the treatment of SAAs and SAPs with the positioning of the stent graft. Therefore, against this background, we performed a comprehensive literature review in order to obtain a more precise insight into the effectiveness of such treatments, making the available evidence more accessible to decision-makers in the real-world clinical practice.

## 2. Materials and Methods

The MedLine (PubMed.gov, U.S. National Library of Medicine, National Institute of Health) and Cochrane databases were searched by three independent authors (OB, AL, and AP) from 1 January 2020 to 31 December 2023 for manuscripts reporting data about SAAs and SAPs treated with stent-graft positioning.

The search terms used were “splenic artery aneurysm”, “splenic artery pseudoaneurysm”, “true aneurysm of the splenic artery”, “false aneurysm of the splenic artery”, and “stent graft”. We considered only original research articles including observational or clinical trials, case reports, and case series. Both emergent and elective cases were included.

Inclusion criteria were: (1) full text in English; (2) adults patients (>18 years-old) affected with both true or false aneurysm of the splenic artery; (3) patients underwent only primary stenting of the splenic artery lesion; and (4) sufficient data about the setting, type of stent-graft used, and the technical and clinical success achieved.

**Figure 1:**
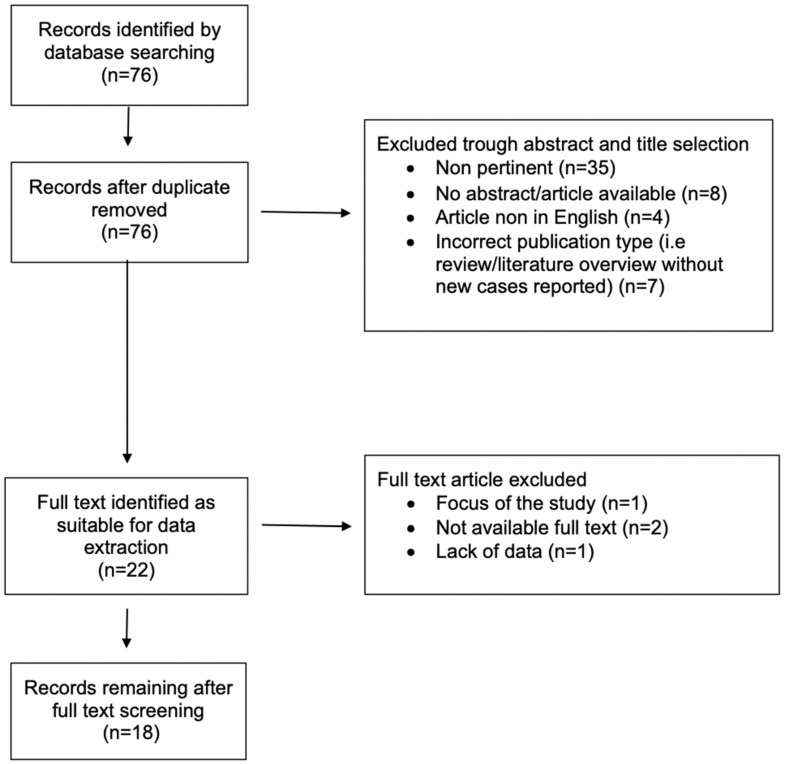
Comprehensive literature review through MedLine and Cochrane database (2020–2023): papers selection.

Articles without an available full text or excessive missing data were excluded (Figure 1). All cases of assisted stenting with coils embolization or flow-diverting devices implantation were excluded. Case reports and other types of studies reporting data on aneurysms affecting other visceral vessels or animal studies were ruled out from the analysis.

The titles and abstracts were reviewed for appropriateness.

Then, the reviewers extracted the data from each study using a predefined database form that included the following information: author’s name, year, and type of study; clinical (symptoms at initial presentation, underlining diseases) and imaging data (aneurysm size and location) from computed angiotomography, arterial Doppler, or magnetic resonance imaging; the type of stent graft used; the complications (end-organ ischaemia, stent-graft migration/kinking/thrombosis, and target vessels or access site rupture/dissection) and outcomes (clinical and technical success, endoleak, and sac enlargement or reperfusion).

### 2.1. Quality Assessment

The studies were analysed in terms of design, heterogeneity, and possible bias. As there were no randomized studies, nor clinical trials, case reports and case series were assessed according to the CARE guidelines and the Joanna Briggs Institute (JBI) Critical Appraisal Checklist for Case reports/studies and for Case Series [10,11,12,13]. Only studies which were well-documented, scientifically rigorous (in terms of completeness, transparency, and data analysis), and followed ethical practices under the CARE guidelines were considered for the analysis. Studies conducted using unethical practices were excluded.

### 2.2. Statistical Analysis

SPSS statistical software (version 25) was used for analysis. Categorical data are presented as counts and percentages, and continuous variables as mean and range.

Because the number of cases was too small and reported data were too heterogenous, no metanalysis to measure the pooled clinical results was performed. A systematic narrative synthesis was performed to summarize and explain the characteristics and findings of the included studies and explore the relationship and findings both within and between the included studies. Data synthesis was conducted independently by the three independent reviewers.

## 3. Results

### 3.1. Demographics, Clinical, and Anatomical Details

Eighteen papers were selected as pertinent for the analysis according to the criteria reported above, totalling 41 patients (n = 20 male 48.8%, mean age 55.5, range 32–82 years).

Most of included patients were affected with SAA (n = 31, 75.6%), and mean aneurysm diameter in non-ruptured cases was 35 mm (range 20–67 mm).

Most of lesions were located at the proximal third of the splenic artery (n = 19/36, 52.8%), but the site of the SAA/SAP was non-reported in five cases.

Clinical presentation and suspected aetiology of SAAs and SAPs are reported in Table 1 and Table 2.

### 3.2. Procedural Details

Reasons for selecting an endovascular approach to treat SAAs/SAPs were reported to be one or more of the following, including: obesity, reiterative abdominal surgery, severe comorbidities, or patients’ preference. None of the selected papers reported the indication for stenting versus simple coil embolization.

Overall, n = 31 (75.6%) procedures were performed in an elective setting, while n = 10 (24.3%) cases were emergent.

A self-expandable stent was the preferred graft with 61% (n = 25) of patients treated with Viabahn (W. L. Gore & Associates, Inc., Flagstaff, AZ, USA) implantation (Table 3).

No data were available about the rate of oversizing applied to the stent graft during the planning of the procedure, nor about the criteria used to select the length of the landing zone.

The preferred vascular access for the endovascular stenting was reported to be the femoral artery, while brachial artery was used only in selected cases to establish a through-and-through access and gain additional support when placing the introducer in a tortuous vessel or in patients presenting with down-going angles of origin in the proximal celiac axis.

Intraprocedural anticoagulation was given in all cases, while data about the medical treatment used postoperatively (single/double antiplatelet or anticoagulation) were mostly lacking.

### 3.3. Technical and Clinical Outcomes

Immediate clinical and technical success was achieved in all cases (n = 37, 90.2%), but n = 4 patients, including one case of intraoperative stent-graft migration, two cases of inability to deliver the stent graft due to extremely tortuous anatomies, and one case of intraoperative splenic artery dissection requiring coiling.

Clinical and technical success were reported regardless the type of stent-graft used.

No cases of arterial or aneurysm rupture nor bleeding were described. There were no procedure-related deaths, but one (2.4%) patient died in hospital from septic shock. Overall, n = 2 (4.9%) patients experienced a splenic infarction that was conservatively managed.

### 3.4. Follow-Up Data

None of the authors of the included papers described the institutional protocol for clinical and imaging surveillances postoperatively, except Reed and colleagues [11], who scheduled a regular follow-up with clinical examination and computed tomography imaging within 4 to 6 months after the procedure and yearly thereafter.

Mean follow-up ranged between 2 days and 3 years, and at the last available imaging control, the complete exclusion of the aneurysm was confirmed in all successfully treated cases (n = 36/41, 87.8%), with one case (n = 1/36, 2.7%) of late stent migration reported 3 years after the initial treatment that was left untreated due to the poor prognosis of the patient.

Three cases (7.3%) of late stent thrombosis (1 partial and 2 complete) were also detected between 3 and 45 months from the initial procedure and left untreated as they were not leading to any clinical condition nor symptom (Table 4).

No cases of an aneurysm growing nor endoleak were reported, and none of the patients required reintervention during follow-up.

## 4. Discussion

### 4.1. Epidemiology and Risk Factors

True and false aneurysms of the visceral arteries are rare entities representing 5% of all intra-abdominal aneurysms overall.

They are mostly incidentally detected. The prevalence of SAAs in general population is low (<1%), and SAPs are even more infrequently reported, with an increased rate of diagnosis in recent years due to the widespread diffusion of more sophisticated and sensitive abdominal imaging, including magnetic resonance imaging (MRI), magnetic resonance angiography (MRA), computed tomography (CT), and CT angiography (CTA).

Nevertheless, SAAs and SAPs are clinically relevant, as they might be associated with a potential rupture and fatal prognosis. Indeed, the mortality rate reaches about 10% of cases diagnosed upon rupture, and prognosis is even poorer for emergent cases in pregnant women, for which the reported mortality may be up to 100% of cases [4].

SAAs are normally degenerative or atherosclerotic aneurysms, but several conditions may be associated with their development (i.e., fibromuscular dysplasia, collagen vascular diseases, and inflammatory conditions). Also, other rare inherited diseases may increase the risk to develop SAAs, making mandatory genetic testing n patients with multiple aneurysms or aneurysms in different locations [4].

Conversely, SAPs are most commonly encountered following infection, abdominal trauma, or iatrogenic injury (for instance, due to arterial catheterization, biopsy, or surgery), or may be related to local inflammatory processes (acute or chronic pancreatitis, peptic ulcer disease, etc.) [7]. They develop following the disruption of the vessel wall with a breach in the intima, and contain bleeding by the tunica adventitia or the surrounding perivascular soft tissue [2,4].

### 4.2. Treatment Strategies

Because of their overall low incidence and prevalence, the natural history of such lesions is relatively poorly defined. Additionally, it is currently unclear which factors are associated with increased risk of rupture or other complications. Therefore, current available guidelines suggest a relatively aggressive approach aiming to prevent aneurysm expansion and potential rupture [4].

The optimal treatment of SAAs and SAPs should be customized on an individual basis considering anatomical and clinical factors, including the underlying comorbidities of the patient and the setting for treatment. Indeed, interventional strategies may vary according to the type of lesion (fusiform versus saccular aneurysm), its size, location, and adhesion to surrounding organs, the clinical setting of diagnosis (ruptured SAA or SAP in a haemodynamically unstable patient versus an asymptomatic/incidentally discovered lesion), and the anatomy of the splenic artery and its collaterals.

Before the widespread diffusion of endovascular approaches, open surgical intervention was considered the gold standard in treatment of SAAs and SAPs. This approach may vary according to the location of the lesion. When it is in the proximal third of the artery, surgery normally involves the simple resection of an aneurysm with or without an interposition bypass, as the collateral flow to the spleen is maintained by the short gastric arteries; otherwise, total splenectomy may be needed for aneurysm located at the hilum of the spleen. Splenectomy could also represent the only viable option in emergent cases when is impossible to achieve the control of the haemorrhage with a simple ligation [3].

Traditional open repair via laparotomy has lately evolved in laparoscopic techniques that have the potential advantage to allow for a faster recovery and, therefore, a shorter hospital stay. Moreover, laparoscopy approach seems to be particularly indicated in pregnant patients because it guarantees lower risk of pre-term labour thanks to the minimal manipulation of intra-abdominal organs [5]. However, surgical approaches might display an increased risk of complications and might be particularly challenging on a technical standpoint in case of compromised hemodynamic status or extensive intra-abdominal inflammatory process.

In recent years, the remarkable improvement of vascular imaging techniques and strategies has allowed us to identify earlier asymptomatic lesions and consequently has promoted more cases of elective treatment of SAAs and SAPs. Endovascular interventions have became the preferred procedures as they also are the most cost-effective strategy and seem to be associated with higher quality of life postoperatively [4].

Embolization represents a widely used approach in the treatment of both SAAs and SAPs, and may be performed through an endovascular (which is also the most common) or a percutaneous approach (either under ultrasound or CTA guidance). The latter is normally performed when endovascular techniques have failed, to treat pseudoaneurysms not accessible endovascularly, or when the lesion is surrounded by solid organ [29,30].

Embolization may be achieved using liquid embolic agents to thrombose the inflow and outflow arteries, vascular plugs, or by filling the sac itself with coils or micro-coils that currently represent the most used material.

More frequently, a sac packing with coils and micro-coils is performed, especially in treatment of saccular pseudoaneurysms with a narrow neck [30]. Although there exist clear immediate benefits (local anaesthesia, shorter hospital stay, and faster recovery) in the use of this approach, end-organ ischaemia, migration of embolization materials into the visceral arteries, aorta, or gastrointestinal tract with possible non-target vessels embolization and post-embolization syndrome (with ongoing pain, fevers, and other systemic symptoms) may occur. Additionally, coil embolization requires a normal coagulation profile, and its main drawbacks when compared with open surgery are a relatively higher rate of failure due to the late reperfusion of the sac (described in up to 30% of cases) and the possible occurrence of intra-procedural or late pseudoaneurysm rupture [30].

**Figure 2:**
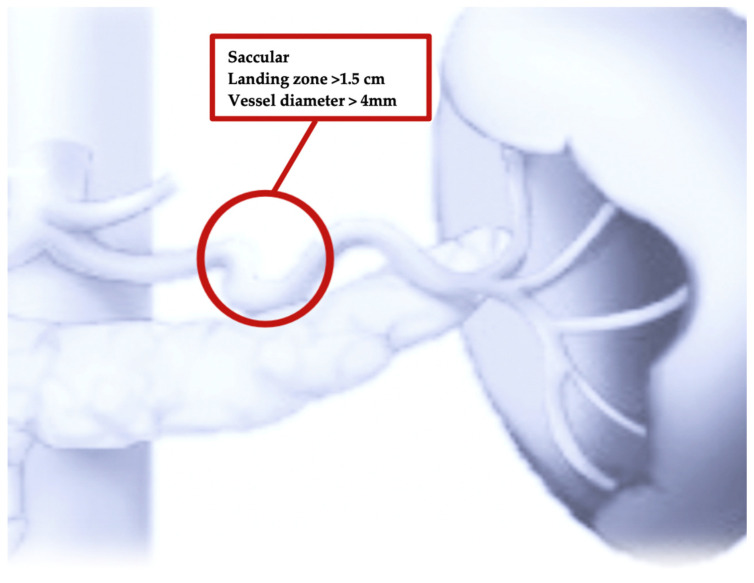
Stent-graft positioning may represent an alternative option, particularly for saccular lesions of the mid splenic artery. Extremely tortuous splenic arteries or those with short landing zones are contraindicated for stent-graft positioning.

Therefore, currently, thanks to the evolution and improvement in endovascular technology and material, stent-graft positioning may represent an alternative option, particularly for saccular lesions of the mid splenic artery (Figure 2). Indeed, the availability of smaller-profile stent grafts, which can be delivered using small sheaths (6F to 8F) over a 0.018-inch or 0.035-inch guidewire system, also make it possible to navigate in smaller vessels with tortuous anatomies.

### 4.3. Preoperative Planning

Optimal imaging favours a detailed and effective interventional planning preoperatively. This should be based on CTA imaging acquired in non-enhanced, arterial, and portal-venous phases and postprocessed with dedicated software before the intervention.

The size, location, extension of the aneurysm, and the collateral branches of the lesion should be studied in multiplanar reconstruction (MPR), and the proximal and distal diameter of the target vessel should be analysed to identify the optimal landing zone for the stent-graft positioning, being aware that measures could be underestimated in cases of actively bleeding SAPs or in cases of vasoconstriction during hypovolemic shock.

Imaging findings will also allow us to plan ahead for the endovascular material to be used in a case-by-case basis; it specifically helps in selecting the more appropriate vascular access, the type and length of introducer sheath, and the catheters and guidewires to be used during the procedure.

### 4.4. Outcomes

The main goal of the present review was to investigate the results achieved with stent-graft positioning in the treatment of SAAs and SAPs. Indeed, we aimed to provide a more precise insight into the effectiveness of such treatment to help define a more clear decision-making process for treating physicians facing this uncommon condition in the real-world clinical practice.

Despite available data coming from individual cases or small series with only short-term results, the analysis of the current literature showed satisfactory results with this approach both in emergent and elective settings. However, the potential wider applicability of this technique should be further investigated in randomized or prospective clinical studies, as the limited number of participants and observational nature of the included studies limit the applicability of our findings to a broader population of patients.

Most of the patients included in the analysis were successfully treated, and no cases of aneurysmal rupture nor reperfusion were reported. These are conversely well described complications following coil embolization, for which the risk of rebleeding occur in up to 12% of cases from collateral vessels and reach up to 30% of cases due to aneurysm recanalization [4,29,30,31].

Recanalization seems to be especially encountered following coil or thrombin embolization of saccular aneurysms, for which sac thrombosis may not be sufficient to protect the treated lesion from pressure transmitted through the thrombus, leading to the potential progression of the disease, growth of the aneurysm’s sac, and even rupture [30].

Therefore, albeit more challenging, it seems that stent-graft positioning might have several potential advantages in respect to coil embolization, including an easier imaging follow-up by avoiding the typical coils artifact, the preservation of distal flow with reduced risk for spleen ischemia (of note, the overall rate of splenic infarction in this review was 4.6%), and hemodynamic advantages in patients affected with portal hypertension [8].

Nevertheless, one of the main limitations of the currently published literature is the lack of data on the long-term results (both in terms of durability, clinical and technical complications, and the need for reintervention) achieved with stent-graft positioning, making it difficult to definitively indicate this approach as durable or applicable without limitation or reservations to all anatomically eligible patients presenting with SAA/SAP.

### 4.5. Complications

As may happen during all endovascular procedures, access to site-related complications such as vessel rupture, dissection, and occlusion can occur [8,14].

Non-target vessels injuries can also be performed during selective catheterization so that operators should have experience with the use of covered and bare-metal stents for managing a rupture or flow-limiting dissections.

In the published literature, no case of rupture of the splenic artery was reported during or after stent-graft positioning. Despite this rare complication, the results of an accidental perforation of the vessel wall by the guidewire tip can be managed at first with temporary balloon occlusion to bridge the timespan until definite surgical treatment.

Stent migration was reported in 7.3% of cases both immediately during the procedure or later during the follow-up period. Underlining causes of this phenomenon are difficult to investigate, even if migration could be possibly related to a non-appropriate oversizing during the preoperative planning, as typically might happen in treatment of acute and emergent cases [16,26]. In such cases, the deployment of self-expanding bare metal stent (i.e., Wallstent, Boston Scientific) across the stent graft was reported to be a successful bailout procedure [16].

Finally, late partial or complete stent thrombosis could possibly occur and can lead to a more or less extensive and clinically relevant spleen infarct that could normally be only conservatively managed [16,24,28].

### 4.6. Specific Consideration for Stent-Graft Positioning

It is worthwhile to underline that not all patients are eligible for stent-grafting of lesions affecting the splenic artery, as several clinical and anatomical criteria should be considered. For instance, the analysis of the data currently available in the literature revealed some general contra-indications to the use of stent-graft in the treatment of SAAs and SAPs:

- Stent-graft positioning should be avoided in extremely tortuous splenic arteries, as the risk to fail in delivering the graft is high [8].
- Stent grafting is not recommended in treatment of lesions with a short landing zone (<1–1.5 cm), as is the case in SAAs or SAPs located in the distal third of the splenic artery or in small-calibre arteries (<4 mm) [8].
- The use of stent graft to treat infected pseudoaneurysms is described, but is controversial. Ouchi and colleagues [3] reported satisfactory results at one year with the use of stents in a septic patient. However, the experience gained with infection of stents placed in other districts suggest applying more caution in this setting. Indeed, we believe an open surgical approach remains the optimal strategy in the setting of mycotic aneurysms, while stenting in association with long-term antibiotic treatment and percutaneous drainage of collections should be restricted to treat selected or unfit patients.

From a technical point of view, self-expandable stent-grafts (i.e., iCast, Atrium, a Maguet Getinge Company, Hudson, NH, USA; Viabahn, W.L. Gore, Flagstaff, AZ, USA, or Fluency, Bard, Tempe, AZ, USA) seem to represent the best option in the treatment of SAAs and SAPs thanks to their flexibility and the accuracy of their delivery system.

Moreover, these devices may be used also in monorail systems, improving manoeuvrability and control in cases of extremely tortuous and small splenic arteries. The main concerns still remain the possible occlusion of a critical side branch, the adequacy of the length of the proximal and distal seal zones for the covered stents, and the limited dimensions of the arteries that can be reasonably treated (5–12 mm). Indeed covered stents have a large profile in comparison to vessel calibre and the difficult placement of the covered stent across the aneurysm neck, which can stimulate artery vasoconstriction, particularly in the splenic artery, making the procedure even more challenging [28]. That is why experienced hands and extensive knowledge of the endovascular material is required.

### 4.7. SAAs in Vasculitis

Vasculitis is a broad group of non infectious disorders that cause inflammation of vessels and mostly affect large and medium-sized arteries and lead to stenoses, aneurysm formation, dissections, or thrombosis [32].

Interventional treatment of inflammatory splenic artery aneurysms in the setting of vasculitis (including Takayasu arteritis, giant cell arteritis, systemic lupus erythematosus, polyarteritis nodosa, and segmental arterial mediolysis and hepatitis-associated vasculitis) or collagenous diseases (Marfan or Ehler–Danlos syndrome) represents a unique challenge.

Historically, endovascular treatment of such lesions was avoided because it has been associated with poor outcomes in terms of patency, but the evolution of material and the use of stent grafts over uncovered stents may mitigate the risk of in-stent restenosis and occlusions that have been frequently reported previously [33].

Corticosteroid therapy still remains the mainstay of treatment for SAAs occurring in inflammatory vasculitis, despite being associated with an increased rate of rupture in mycotic or atherosclerotic aneurysms [4,32].

### 4.8. Surveillances and Long-Term Medical Treatment

Coils are highly radio-dense materials leading to artefacts at the CTA. Therefore, imaging surveillance of patients treated with embolization should be performed with duplex ultrasound or an MRI [4].

Conversely, follow-up schedule and imaging modality is yet to be defined for the stenting of aneurysmal splenic arteries, but continued monitoring is imperative, as long-term results have not been fully investigated and reported, and most of treated patients are relatively young.

In addition, taking into account that after angioplasty and stenting for mesenteric arterial stenoses or occlusion imaging control is normally recommended after 1, 6, and 12 months and yearly thereafter, we suggest this same frequency could be applied for surveillance in stented SAAs and SAPs [34].

The same rationale could be used when deciding about the antiplatelet (single versus double) or anticoagulation treatment to be performed during the follow-up period [34]. Indeed, when the use of these drugs is balanced against the risk related to the clinical conditions and comorbidities of the treated patient, anti-coagulation/antiplatelet therapy might increase the patency rate and prevent the late occurrence of spleen infarct, avoiding stent-graft occlusion or thrombosis that represent a non-insignificant aspect to be considered when using small diameter stents.

### 4.9. Future Perspective

In the latest year, the evolving technology of flow-diverter devices (FDDs) that have been originally developed for repairing intracranial aneurysms may provide another tool to allow for the effective exclusion of an aneurysm while preserving the parent vessel.

FDDs divert the blood flow that became stagnant into the aneurysm’s sac until complete thrombosis, which determines the development of a new endothelium that covers the aneurysm ostium, excluding it from the circulation [35]. This may represent a valuable option for SAAs, but since the thrombosis of the lesion occurs too slowly with a possible rupture in the interim, FDDs are normally inefficacious in treatment of pseudoaneurysms [30].

Their use in extracranial vasculature is off-label and the costs are still high, but they display the potential advantages of enhanced flexibility and trackability in respect to currently available covered stents, and are particularly indicated in treatment of small-calibre arteries thanks to their high navigability [35].

## 5. Conclusions

Endovascular techniques are the preferred option in treatment of SAAs/SAPs whenever technically possible, thanks to their reduced invasiveness.

When specific anatomical criteria are respected, stent-grafts display potential advantages when compared to simple coil embolization, being at the same time efficacious in excluding the aneurysm while preserving distal blood flow to the spleen.

In conclusion, despite the reported results being promising, the level of evidence to guide clinical decision-making is low, and a more precise definition of the anatomical and technical criteria required for stenting of the splenic artery is needed.

The inherent bias in case reports and series on which the current review is based make it impossible to give any conclusive recommendations based on this limited evidence.

Further large scale observational studies with long-term follow-up are needed to provide more robust data to contemplate the wider applicability of this approach in the foreseeable future.

## 6. Limits

This study is inherently limited by the non-standardized nature of case reports that were selected for the analysis, impacting on the uniformity of the variables of interest. Additionally, the extracted data were obtained from published case reports which span over 20 years, encompassing several evolutions of technical tips and materials used.

## Figures and Tables

**Table 1:** Demographic, anatomical, and clinical details of included patients.

	N Patients 41 (100)
Sex	
Male	20 (48.8)
Female	21 (51.2)
Age	55.5 (range 32–82 y)
Type of lesion	
SAA	31 (75.6)
SAP	10 (24.4)
Mean aneurysm diameter (non-ruptured cases)	35 mm (range 20–67 mm)
Location	
Proximal third of the splenic artery	19 (46)
Middle third of the splenic artery	15 (36.6)
Distal third of the splenic artery	2 (4.9)
Non-reported	5 (12.2)
Aetiology	
Cirrhosis/pancreatitis	4 (9.7)
Postoperative	4 (9.7)
Spontaneous dissection	1 (2.4)
Idiopathic	1 (2.4)
Unknown/non-reported	31 (75.6)
Clinical presentation	
Asymptomatic/incidentally discovered	22 (53.6)
Fever	1 (2.4)
Pain	4 (9.7)
Rupture (haemorrhagic shock/gastrointestinal bleeding)	3 (7.3)
Non-reported	11 (26.8)
Setting for repair	
Emergent	10 (24.3)
Elective	31 (75.6)

Data are reported as n (%) or mean/range.

**Table 2:** Clinical and anatomical details.

Author, Year, Study Type	Patient and Age	SAA/SAP	Size/Location	Setting	Symptoms	Aetiology
Yoon et al., 2001, case report [9]	Male, 50 years	SAA	28 mm, proximal third	Elective	Asymptomatic	Cirrhosis Pancreatitis
Arepally et al., 2002, case report [14]	Female, 63 years	SAA	25 mm, middle third	Elective	Asymptomatic	Cirrhosis and Portal hypertension
Larson et al., 2002, case report [15]	Female, 50 years	SAA	20 mm, middle third	Elective	Asymptomatic	Unknown
Brountzos et al., 2003, case report [16]	Female, 66 years	SAP	35 mm, proximal third	Elective	Asymptomatic	Pancreatitis
Karaman et al., 2005, case report [17]	Male, 56 years	SAA	28 mm, proximal third	Elective	Pain	Unknown
Guller et al., 2006 [18]	Female, 55 years	Saccular SAA	26 mm, middle third	Elective	Fever	Unknown
Rossi et al., 2008, case series [19]	3 Female, (mean age 62 years, range 55–76)	SAA	35 mm, middle third 30 mm, proximal third 30 mm, middle third	Elective	Asymptomatic	Unknown
Kim et al., 2009, case report [20]	Male, 82 years	Saccular SAA	67 mm, distal third	Elective	Asymptomatic	Unknown
Briard et al., 2009 [21]	Female, 47 years	SAA	35 mm, proximal third	Elective	Asymptomatic	Portal hypertension
Xin et al., 2011, case report [22]	Female, 36 years	Ruptured SAP	20 mm, proximal third	Emergent	Asymptomatic	Following abdominal surgery
Go’es et al., 2012, case report [23]	Female 64 years	SAA	65 mm, proximal third	Elective	Asymptomatic	Portal hypertension
Kunzle et al., 2013, case report [24]	N = 3 (2 males, 1 female) mean age 60 years (40–82 years)	2 SAP; 1 SAA	NR	2 Emergent 1 Elective	Pain in 2 cases asymptomatic in 1 case	2 postoperative; 1 idiopathic
Guang et al., 2015, case report [25]	Female 54 years	SAA	28 mm, middle third	Elective	Rupture	Postoperative
Reed et al., 2015, case series [8]	N = 10 (4 males, 5 females) median age 64 years (range 42–77 years)	9 SAA 1 SAP	Median aneurysm diameter was 2.8 ± 1.3 cm (range, 2.0–5.7 cm). Proximal third 2; middle third 7; distal third 1	9 Electives 1 Emergent	9 asymptomatic 1 gastrointestinal bleeding	NR
Rebonato et al., 2016, case series [26]	Male, 73 years	SAP	60 mm, proximal third	Emergent	Pain	Unknown
Anton et al., 2017, case serie [27]	N = 2 (2 males) 50 y and 81 years	SAA	39 mm and 21 mm, NR	Elective	Asymptomatic	NR
Venturini et al., 2018, case series [28]	N = 11 (7 male, 4 female) mean age 58.3 years (range 32–70 years)	7 SAAs; 4 SAPs	NR, 3 middle third; 8 proximal third	4 Emergency 7 Elective	NR	NR
Ouchi et al., 2018, case report and literature review [3]	Male, 43 years	Infected ruptured SAP	64 mm, proximal third	Emergency	Haematemesis	Spontaneous dissection

SAA Splenic Artery Aneurysm, SAP Splenic Artery Pseudoaneurysm; NR Non-Reported.

**Table 3:** Technical details and outcomes.

Author, Year	Device	Technical Clinical Success	Clinical Success	Complications	Follow-Up
Yoon et al., 2001 [9]	Graftmaster	Y	Y	None	3 months
Arepally et al., 2002 [14]	Wallgraft	Y	Y	None	2 weeks
Larson et al., 2002 [15]	Wallgraft	Y	Y	Groin hematoma	12 months
Brountzos et al., 2003 [16]	Viabahn	Stent graft migration treated with Wallstent placement	N	Splenic infarction	1 months
Karaman et al., 2005 [17]	Wallgraft	Y	Y	None	2 months
Guller et al., 2006 [18]	iCAST	Y	Y	None	2 days
Rossi et al., 2008 [19]	Graftmaster in 2 cases; iCAST in 1 case	Y	Y	One case of Splenic infarction	Mean 20 (range 18–24 months)
Kim et al., 2009 [20]	Graftmaster	Y	Y	None	18 months
Briard et al., 2009 [21]	Fluency	Y	Y	None	2 months
Xin et al., 2011 [22]	Fluency	Y	Y	None	48 months
Go’es et al., 2012 [23]	Viabahn	Y	Y	None	1 months
Kunzle et al., 2013 [24]	Graftmaster in 2 cases and Fluency in 1 case	Y	Y	1 thrombosis between 5 and 12 months following stent placement	NR
Guang et al., 2015 [25]	Fluency	Y	Y	None	12 months
Reed et al., 2015 [8]	Viabahn (+ EverFlex in 2 cases)	Y (8 patients) The two technical failures occurred due to inability to properly deliver the stent graft in patients with very tortuous anatomies	Y	Brachial artery occlusion dissection: excision of the intimal flap, thrombectomy, and patch angioplasty. One case of a small peripheral wedge-shaped asymptomatic infarct.	Mean 9 months (14 days–57 months)
Rebonato et al., 2016 [26]	Viabahn	Y	Y	Stent graft occlusion (3 months postoperatively) and stent graft migration within the stomach (3 years postoperatively)	36 months
Anton et al., 2017 [27]	E-ventus BX	Y	Y	none	Mean 13 months (range 1–26)
Venturini et al., 2018 [28]	Viabahn	Y (10/11)	9/11 (1 death septic shock and 1 SA dissection requiring coil embolization)	Partial intrastent thrombosis after 45 months	Mean 39.2 months (Range 1–127 months)
Ouchi et al., 2018 [3]	Viabahn	Y	Y	None	12 months

Y = yes, N = no; NR = Non Reported. Viabahn (W. L. Gore & Associates, Inc., Flagstaff, AZ, USA), Graftmaster (Abbott Vascular, Santa Clara, CA, USA), Fluency (Bard Peripheral Vascular, Inc., Tempe, AZ, USA), Wallgraft (Boston Scientific, Inc., Watertown, MA, USA), iCAST (Atrium, Hudson, NH, USA), E-ventus BX balloon-expandable stent graft system (Jotec, Hechingen, Germany); EverFlex (Medtronic, Minneapolis, MN, USA).

**Table 4:** Clinical and technical outcomes during in-hospital period and follow-up.

	N Patients 41 (100)
Intraoperative data	
Immediate technical success	37 (90.4)
Intraoperative stent migration	1 (2.4)
Inability to deliver the stent-graft	2 (4.9)
Artery dissection requiring coiling	1 (2.4)
Procedure-related death	-
In-hospital complication	
Splenic infarction	2 (4.9)
In-hospital mortality	1 (2.4)
Follow-up data	
Late stent thrombosis	3 (7.3)
Endoleak/aneurysm growing	-
Reintervention	-
Overall Mortality	1 (2.4)

Data are reported as n (%).

## Data Availability

The data presented in this study are available on request from the corresponding author.
